# Spectacles; Their Uses and Abuses in Long and Short Sightedness; and the Pathological Conditions Resulting from Their Irrational Emplopment

**Published:** 1851-01

**Authors:** 


For sale by Phinney & Co.

---

*Spectacles; their uses and abuses in long and short sightedness; and the pathological conditions resulting from their irrational employment,* By J. Sickel, M. D., of the Faculties of Berlin and Paris, Ciln. Prof. of dis-diseases of the eye, etc., etc., etc. Translated from the French by permission of the author, by Henry W. Williams, M. D., Fellow of the Mass. Med. Society, etc. Boston, Phillips, Sampson, & Co., 110, Washington street. 1850. 8 Vol., pp. 202.

As the translator remarks in his preface to this treatise, the catalogue of our medical literature contains no work, on the uses and abuses of spectacles, prior to this publication. And that, in this particular, a *hiatus* existed, no one can doubt, who has given any attention to the subject, and, more especially, one who, for reasons of a personal nature, has been obliged to bestow some thought upon it. Practitioners of medicine are often consulted respecting the propriety of wearing spectacles in cases of myopia, presbyopia, etc., and their judgment is solicited as to the choice of glasses, and other points bearing on this method of giving artificial assistance, or protection to the organs of sight. Patients have a right to look to the Profession for this kind of information; but there is reason to believe that they are not infrequently disappointed in obtaining it, if, indeed, they are not sometimes misled by the enunciation of incorrect notions.

The subject of myopia, as affecting the young, is one which concerns not alone the visual organs. The mind and character of an individual may be influenced, in no small degree, by this defect. It tends to repress the observing powers by diminution of the amount of stimulating incentives to their exercise, and thus indirectly to favor an undue development of the meditative faculties. This is a point of view in which we do not remember to have seen this disorder of vision considered, but we have no doubt of the correctness of the remark. Here, then, is an additional reason why this difficulty should receive proper attention in early life, before the natural tendency to modify the intellectual character of the individual has had time to produce mental habits and effects which it will be difficult afterward to overcome.

Myopia, in a large proportion of cases, whether congenital, or acquired in early life, (the latter being very frequently the case,) is susceptible of being remedied if discovered, and the proper measures resorted to. The latter are of an obvious character, consisting in the habitual exercise of vision upon distant objects, on the one hand, and avoiding, on the other hand, the use of the eyes in observing objects at too near a focus. Occupations and pursuits are to be selected and pursued in reference to this defect. Spectacles are not to be resorted to while there is reason to hope for success in overcoming the defect. When it becomes necessary to resort to them› certain principles are to be observed in the selection of to glasses. We quote on this topic from Dr. Sichel's work:—

"When concave glasses are judiciously selected, they ought neither to lessen or bring objects near, nor produce a too great distinctness of vision, which may sometimes even amount to oxyopia, a name given to a peculiar condition where the eye is over-excited and dazzled by the cleaness with which objects are perceived. Still less should the glasses cause a feeling of annoyance, of pressure, of pain in the eyes, of fatigue after using them for some time, or any other abnormal phenomenon. When the spectacles are laid aside, the sight should not become more confused at short distances than it previously was; nor ought a species of shade, or badly defined image, representing either the form of the spectacles or their frame, or the objects which have been observed, to remain before the eyes."

In Presbyopia (far sightedness,) the opposite of myopia, (near sightedness,) the proper adjustment of the degree of convexity in the glasses selected is of great importance, with reference to the preservation of the powers of vision, and the prevention of other ocular affections. The rule is, to select the lowest curve of convexity that will answer the ends desired. One of the evils attending the use of glasses with greater convexity than is needed is, the ability of the eye to accommodate itself to objects at different distances is impaired. We quote the author's remarks on this point:—

"Generally speaking, every person, whether presbytic or myope, can make use of glasses of different curves; only, with the stronger numbers he sees more clearly, and is forced, if presbytic, to bring objects nearer; if myopic, to place them further from the eyes, but without thus causing them to be magnified or dimisished in volume. The more powerful the glass, the less latitude it allows in the position of bodies looked at. The feebler spectacles allow this position to be varied to a certain extent without any notable change in the distinctness of the visual perception; an evident proof that these glasses of slight curvature allow the conservation of a certain degree of the accommodative faculty. An example will make what has been said more intelligible, and will facilitate the comprehension of the conclusions which we shall deduce from it; a presbytic person who has never yet employed spectacles, but has not allowed the opportune moment for their adoption to pass by, can generally read equally well with Nos. 72, 66, and 60; he will, however, find, if he pays strict attention to his sensations, that with the last-named number he will be compelled to bring the book nearer, and to hold it more invariably at the same distance: whilst the first obliges him to place it further off, and permits him to bring it nearer or remove it, within a certain distance, without his sight becoming dim or sensibly fatigued. Those more feeble glasses, then, permit the preservation of a certain degree of the faculty of accommodation, which those of a greater curvature diminish and end by abolishing, in a manner more positive in proportion as their use is more constant. If the eye can make use of several numbers solely by changing the position of the objects, it is because it accommodates itself to the focus of the glasses. Once habituated to a shorter focus, it cannot, without difficulty, return to more feeble glasses; this difficulty, always proportioned to the power of the spectacles, may end by becoming insurmountable when this power has been exercised. Thence results the high importance of the precept we have given, always to choose the most feeble number with which the patient can distinguish clearly and without fatigue, without change of the apparent volume of the objects, or without being forced to place them at a distance very different from that which the unassisted eye admits of. A presbytic who can employ, with nearly equal facility, Nos. 72, 66, and 60, by using the last for some time would accommodate his sight to this number. The modifications which age effects in the visual organ making their inevitable progress, he will be forced to change his spectacles at a given time, and much sooner than would otherwise be requisite, because the accommodatlon of the sight to spectacles of too great power is soon accompanied by fatigue and a want of clearness of vision, like all too continuous exercise of the adjusting faculty, especially at short distances.

He has then again to select among several numbers of which the effect will appear to him to differ very little, as Nos. 54 and 48. But, for the same motive as before, he will infallibly choose the strongest, as being that which apparently renders him the most efficacious aid. Thus the progression is very rapid; the further it descends, the more the glasses destroy the faculty of accommodation, not only during their use, but also during the time when they are laid aside; for, bound as it were to the focus of the spectacles, during all the time they are worn, the sight no longer easily adapts itself to greater distances. Thus the presbytic who employ more feeble numbers can yet read for some time with the naked eye, and preserve all the integrity of their visual range for remote distances, whilst those who constantly read or work with powerful glasses, end by finding themselves unable to dispense with them, and often even can no longer see large objects as far as formerly.

If, then, after having seen perfectly well with spectacles of a certain number, a person uses, without reason, or for experiment, glasses of greater power, he will find, at the end of a few weeks of their employment, that, on returning to the number primarily used, he sees infinitely less well than formerly, if he can see at all; an evident proof that spectacles limit the focus, and diminish the adjustive faculty, which they may end by destroying. That which might, in this respect, be assumed *a priori*, is confirmed, in each point, by experience, which proves, at the same time, that it is of the highest importance to proceed rationally in the choice of spectacles, and to begin, according to the rules we have endeavored to establish, by those of the feeblest power, substituting others, of greater strength, by insensible degrees. Here, as everywhere, the physiological laws forbid sudden transitions, which, by their shock, produce a disturbance, which is almost always injurious to the function."

The foregoing extracts, aside from their intrinsic practical value, will serve as specimens of the style and character of the work.

For sale by Phinney & Co., and Derby & Co.

